# A preliminary study of placental umbilical cord whole blood transfusion in under resourced patients with malaria in the background of anaemia

**DOI:** 10.1186/1475-2875-5-20

**Published:** 2006-03-23

**Authors:** Niranjan Bhattacharya

**Affiliations:** 1 Foetal Cell/Tissue Transplant in Adults, Bijoygarh State Hospital and Moore Avenue Govt. Specialist Polyclinic, Tollygunge, Calcutta 700040, India

## Abstract

**Background:**

Malaria is an annual killer of over one million people globally and its essential co-morbidity is anaemia. Cord blood, because of its rich mix of foetal and adult haemoglobin, high platelet and WBC counts, hypo-antigenic nature, altered metabolic profile and high affinity for oxygen as well as its anti-malarial effect, is an ideal choice in malaria with anaemia, necessitating blood transfusion.

**Methods:**

This paper presents an alternative protocol for fresh whole blood/packed cell transfusion from the hospital's biological waste resources, i.e., the placenta, after the birth of a healthy baby from a healthy mother. This collected blood was routinely transfused to patients admitted in our hospital with severe anaemia in the background of confirmed malaria. 94 units of placental umbilical cord whole blood were collected after lower uterine caesarean section (LUCS) from consenting mothers (from 1^st ^April 1999 to April 2005), and safely transfused to 39 informed, consenting patients (age varying from 8 to 72 years). The collected volume of cord blood from each placenta (Unit) varied from 52 ml to 143 ml, with a mean packed cell volume of 48.9 ± 4.1 SD and a mean haemoglobin concentration of 16.4 Gm percent ± 1.6 Gm percent SD. The blood was immediately transfused after following the standard adult blood transfusion protocol of screening and cross-matching between the donor and the recipient. On occasion, the collected cord blood was preserved in the refrigerator, if no volunteer was readily available, and transfused within 72 hours of collection.

**Results:**

Cord blood transfusion was tested on twenty two patients infected with *Plasmodium falciparum *and 17 patients with *Plasmodium vivax*. For inclusion in this study, the patient's plasma haemoglobin had to be 8 gm percent or less (the pre-transfusion haemoglobin in the malaria-infected patients in this series varied from 5.4 gm/dl to 7.9 gm/dl). The rise of haemoglobin within 72 hours of two units of freshly collected cord blood transfusion was 0.5 gm/dl to 1.6 gm/dl. Each patient received two to six units of freshly collected cord blood transfusion (two units at a time), depending on availability and compatibility. No clinical immunological or non-immunological reaction has been encountered in this series.

**Conclusion:**

Properly screened cord blood is safe for transfusion, in victims of severe malarial anaemia who need transfusion support.

## Background

Malaria, caused by infection with *Plasmodium falciparum*, kills over 1 million people each year [1]. Anaemia due to malaria is a major health problem in endemic areas, particularly for young children and pregnant women. This anaemia is caused by excess removal of non-parasitized erythrocytes in addition to immune destruction of parasitized red cells and impaired compensation for this loss by bone marrow dysfunction. Though *P. falciparum *is the predominant cause of anaemia and its complications, *Plasmodium vivax *can also cause anaemia and thrombocytopaenia requiring hospitalization, although to a much lesser extent.

To combat severe anaemia, several options are available: concentrated fresh red blood cell (RBC) transfusion, erythropoietin injection, blood substitutes (oxygen carriers like perflurocarbon compounds, etc), dietary supplementation of haematinics along with other essential nutrient support needed for proper erythropoiesis.

The problem, however, lies in the availability of properly screened blood, in many areas of the developing world. The cost and complications of erythropoietin therapy, which has fuelled the continued search for an ideal blood substitute, is an added difficulty. In a report of the World Health Organization, it was observed that there are about 500,000 pregnancy-related deaths globally, of which at least 25 percent maternal deaths are due to the loss of blood [2]. An estimated 13 million units of blood worldwide are not tested against human immunodeficiency viruses or hepatitis viruses, and in some developing countries 80 percent of the blood supply comes from paid donors or replacement donors (family friends or acquaintances) even when the virus-infected population is high [3].

Concerns about the safety and adequacy of the blood supply have fostered twenty years of global research into the so-called "blood substitutes" among them the oxygen carriers based on modified haemoglobin.

Foetal haemoglobin is a natural stress response to haemoglobin synthesis, which may be augmented in case of thalassemia by hydoxyurea treatment. Other conditions like pregnancy, diabetes, thyroid disease or anti-epileptic drug therapy, can also increase the foetal haemoglobin concentration. Placenta is an abundant source of foetal haemoglobin and placentas are an unused resource: in India alone, there are more than 20 million placentas produced every year of which 99.9% are discarded.

## Materials and methods

Whether foetal haemoglobin-rich placental umbilical cord whole blood (which has the potential to carry more oxygen to the tissue Vol/Vol than adult blood, because of its foetal haemoglobin component) can be a safe substitute for adult blood, if collected aseptically after the birth of a healthy newborn at or near term, is the main scientific query behind the present study. Human placental umbilical cord blood was collected from consenting mothers aseptically after lower uterine Caesarean section under general or regional anesthaesia. If there was gross prematurity or dysmaturity or the projected weight of the foetus was less than 2 kg, or if the mother was suffering from any specific disease like hepatitis or HIV, etc., the cord blood collection was abandoned. The collection process started only after the baby was safely removed from the operation field. Another sample of the cord blood collected from the placenta was immediately tested for blood group (Rh and ABO), Human Immunodeficiency Virus (HIV 1 and 2), hepatitis B and C, VDRL, malaria, bacterial and fungal infections. When the collection was complete, the blood bag tubing was closed, sealed, and transfused to a malaria victim as early as possible as per standard blood transfusion protocol, as previously reported [4-6]. Host bilirubin, urea, creatinine, glucose, were also tested along with Hb/TC/DC/ESR, before transfusion and the tests were repeated 72 hours after transfusion in some randomly selected cases to see the effect of transfusion on the rise of haemoglobin, as well as the impact on the host's hepato-renal and metabolic profile, using standard methods.

If a patient with confirmed malaria, whose haemoglobin was less than 8 gm/dl, was not readily available, the cord blood was stored between 1°C and 4°C. This blood was transfused within three days of collection to a malaria patient with anaemia as per specifications mentioned earlier, following the World Health Organization (WHO) standard adult blood transfusion guidelines and strictly adhering to the institutional ethical committee rules and the patient consent protocol.

## Results and discussion

39 patients with confirmed malaria were randomly selected for the present study after approval was given by the Institutional ethical committee for each case and the voluntary patient consent protocol was followed. The age of the patients varied from 8 to 72 years, (mean 39.4 years), of whom 24 were male and 15 were female. In this series, twenty-two patients were infected with P. falciparum and seventeen had P. vivax infection.

The 94 units of cord blood (52 ml to 143 ml in volume, mean 81 m1+6.6 ml SD, median 82 ml, mean packed cell volume 48.9 + 4.1 SD, mean haemoglobin concentration 16.4 Gm percent + 1.6 Gm percent SD) were transfused to the 39 informed, consenting patients from 1 April 1999 to April 2005. Two units were transfused at a time to individual patients. The recipient who got the maximum amount received six units of placental blood.

**Figure 1:**
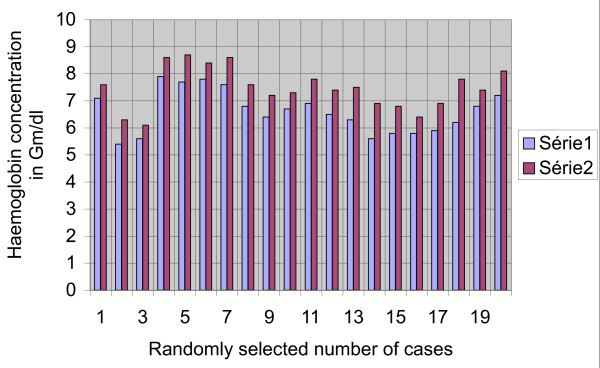
**Graphical impact of 2 units of cord blood transfusion on the host after 72 hours**. Series 1: pre-transfusion haemoglobin in Gm/dl. Series 2: post-transfusion haemoglobin in Gm/dl (after 72 hours).

The pathophysiology [7], iron metabolism [8,9] and erythropoietin production [10] in case of anemia in chronic disease is different. The amount of transfusion depended on the severity of anaemia and the availability of compatible and screened cord blood. The pre-transfusion haemoglobin in the malaria infected patients in this study varied from 5.4 gm/dl to 7.9 gm/dl for falciparum infection and 6.3 gm/dl – 7.8 gm/dl in vivax-infected patients. The rise of haemoglobin as estimated after 72 hours of the transfusion of two units of cord blood was 0.5 gm/dl to 1.6 gm/dl (Figure 1). What is interesting is the fact that there is a slow but sustained rise of haemoglobin on the seventh day after transfusion (series 3).

**Figure 2:**
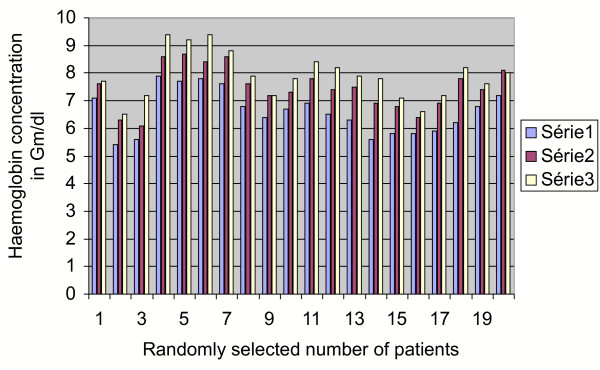
**Graphical impact of 2 units of cord blood transfusion on the host after 72 hours and 7 days**. Series 1: pre-transfusion haemoglobin in Gm/dl. Series 2: post-transfusion haemoglobin in Gm/dl after 72 hours. Series 3: post-transfusion haemoglobin in Gm/dl after 7 days.

**Figure 3:**
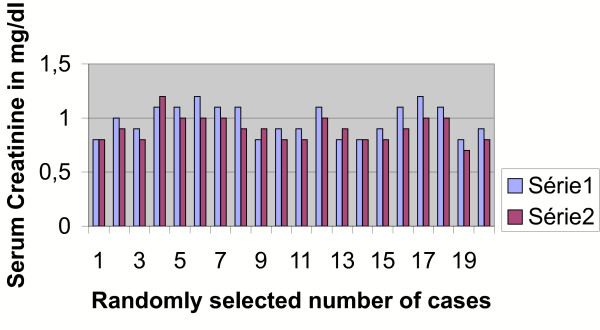
**Graphical impact of 2 units of cord blood transfusion on the host's creatinine level as seen after 72 hours**. Series 1: pre-transfusion Creatinine in mg/dl. Series 2: post-transfusion Creatinine in mg/dl after 72 hours.

**Figure 4:**
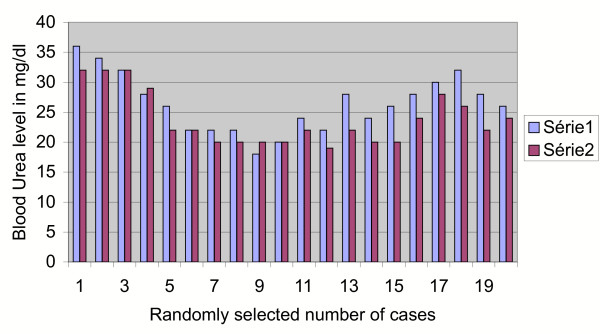
**Graphical impact of 2 units of cord blood transfusion on the host's urea level as seen after 72 hours**. Series 1: pre-transfusion Urea level in mg/dl. Series 2: post-transfusion Urea level in mg/dl after 72 hours.

**Figure 5:**
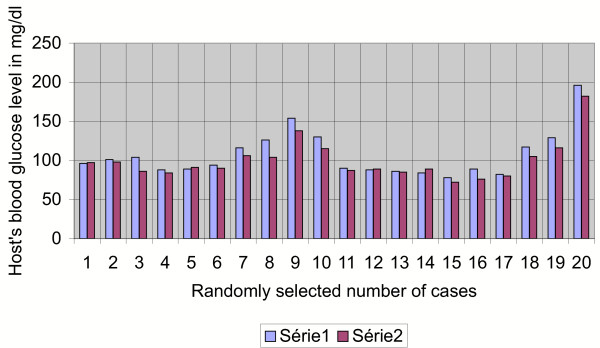
**Graphical impact of 2 units of cord blood transfusion on the host's glucose level as seen after 72 hours**. Series 1: pre-transfusion glucose in mg/dl. Series 2: post-transfusion glucose in mg/dl (after 72 hours).

**Figure 6:**
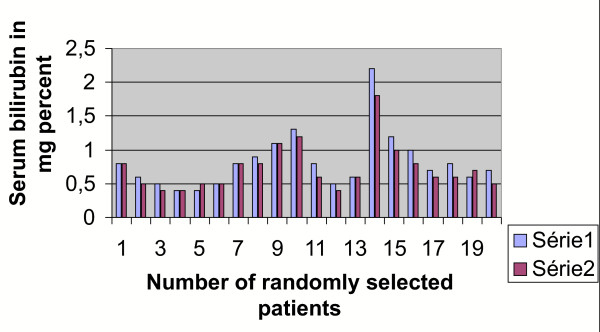
Graphical impact of 2 units of cord blood transfusion on the host's bilirubin level as seen after 72 hours.

A univariate analysis using Fishers' exact test was performed for the results of series 2 (rise of haemoglobin after 72 hours from pre-transfusion value) and series 3 (rise of haemoglobin after 7 days from pre-transfusion value). The difference between Group 2 and 3 values and its comparison with the pre-transfusion haemoglobin appeared to be statistically significant (p < less than .003). This effect could be due to the bone marrow stimulating effect of the different cytokine systems of the placental blood (Figure 2). No immunological or non-immunological reaction or adverse metabolic impact on the recipient has been encountered so far. There was no detected rise of serum creatinine (Figure 3), urea (Figure 4), glucose (Figure 5), bilirubin (Figure 6), on the recipients of two units of cord blood, when compared to the pre-transfusion level. There was also an improvement of appetite and a sense of well being in all the recipients of cord blood transfusion.

## Conclusion

The placenta is a rich source of cord blood (at term there is up to 150 ml of blood in placental circulation). Cord blood is protected from infection as a result of nature's finest biological sieve [11,12], i.e., the placenta and contains 60–80% foetal haemoglobin (which can carry 60% more haemoglobin than adult haemoglobin) and has also a high WBC and platelet content, is hypo-antigenic in nature, and has an altered metabolic profile. It may also have the potential, due to its rich cytokine and growth factor content [13], to play a role in immune response modification in chronic anaemia.

In the developed world, umbilical cord blood (UCB) is now accepted as an alternative source for haematopoietic stem cells for transplantation, especially in children, in view of its many practical advantages. Currently there are about 100,000 units available worldwide [14]. The high oxygen affinity and anti-malarial effect of foetal haemoglobin in cord blood are additional advantages for transfusion in malaria patients with anaemia [15].
